# Hypoparathyroïdie et démence d’installation rapide: penser au syndrome de Fahr

**DOI:** 10.11604/pamj.2018.30.82.15587

**Published:** 2018-05-29

**Authors:** Ines Kechaou, Imène Boukhris

**Affiliations:** 1 Service de Médecine Interne B, Hôpital Charles Nicolle, Tunis, Tunisie

**Keywords:** Syndrome de Fahr, démence, hypoparathyroidie, Fahr syndrome, dementia, hypoparathyroidism

## Abstract

Fahr syndrome is a rare disorder, whose prevalence is lower than 0.5%. Clinical manifestations are variable ranging from mere behavioral disorders to tetany crises and severe neuropsychiatric manifestations. It may be idiopathic or secondary to numerous causes dominated by phosphocalcium metabolism disorders whose main cause is hypoparathyroidism. Positive diagnosis is based on radiology. We report the case of a 78-year old hypertensive female patient with a history of hypothyroidism, hypoparathyroidism after total thyroidectomy performed 30years before. She was treated with Levothyrox® 100 µg 2 capsules/day , One-Alpha® 1 μg 3 capsules/day and calperos® 2 capsules/day resulting in euthyroidia confirmed by clinical examination and laboratory tests and normal phosphocalcic test. Patient's outcome was marked, in 2010, by the development of rapidly worsening cognitive impairment and of early onset dementia in a few months. Neurological examination showed extrapyramidal syndrome. Cerebral CT scan showed large basal ganglia calcifications, white and gray linear substance near the junction, in the brain bulb and in the cerebellum. The diagnosis of Fahr syndrome, revealed by dementia occurred after a history of hypoparathyroidism, was then retained

## Image en médecine

Le syndrome de Fahr est une entité rare dont la prévalence est inférieure à 0,5%. Ses manifestations cliniques sont variables allant des simples troubles du comportement aux crises de tétanie et aux manifestations neuropsychiatriques sévères. Il peut être idiopathique ou secondaire à de nombreuses étiologies dominées par les anomalies du métabolisme phosphocalcique dont la principale cause est l'hypoparathyroïdie. Son diagnostic positif est radiologique. Nous rapportons l'observation d'une patiente âgée de 78 ans hypertendue aux antécédents d'hypothyroïdie, d'hypoparathyroidie post thyroïdectomie totale il ya 30ans. Elle est traitée par levothyrox®100 µg 2 cp/j, un-alpha®1 µg 3cp/j et calperos® 2cp/j avec euthyroidie clinique et biologique et un bilan phosphocalcique sans anomalie. L'évolution a été marquée par l'apparition en 2010 de troubles cognitifs d'aggravation rapide avec installation d'une véritable démence en quelques mois. L'examen neurologique avait objectivé un syndrome extrapyramidal. Le scanner cérébral avait mis en évidence des calcifications étendues des noyaux gris centraux, à la jonction substance blanche substance grise linéaires, du bulbe cérébral et du cervelet. Le diagnostic d'un syndrome de Fahr, révélé par une démence suite à une hypoparathyroidie ancienne, a été alors retenu.

**Figure 1:**
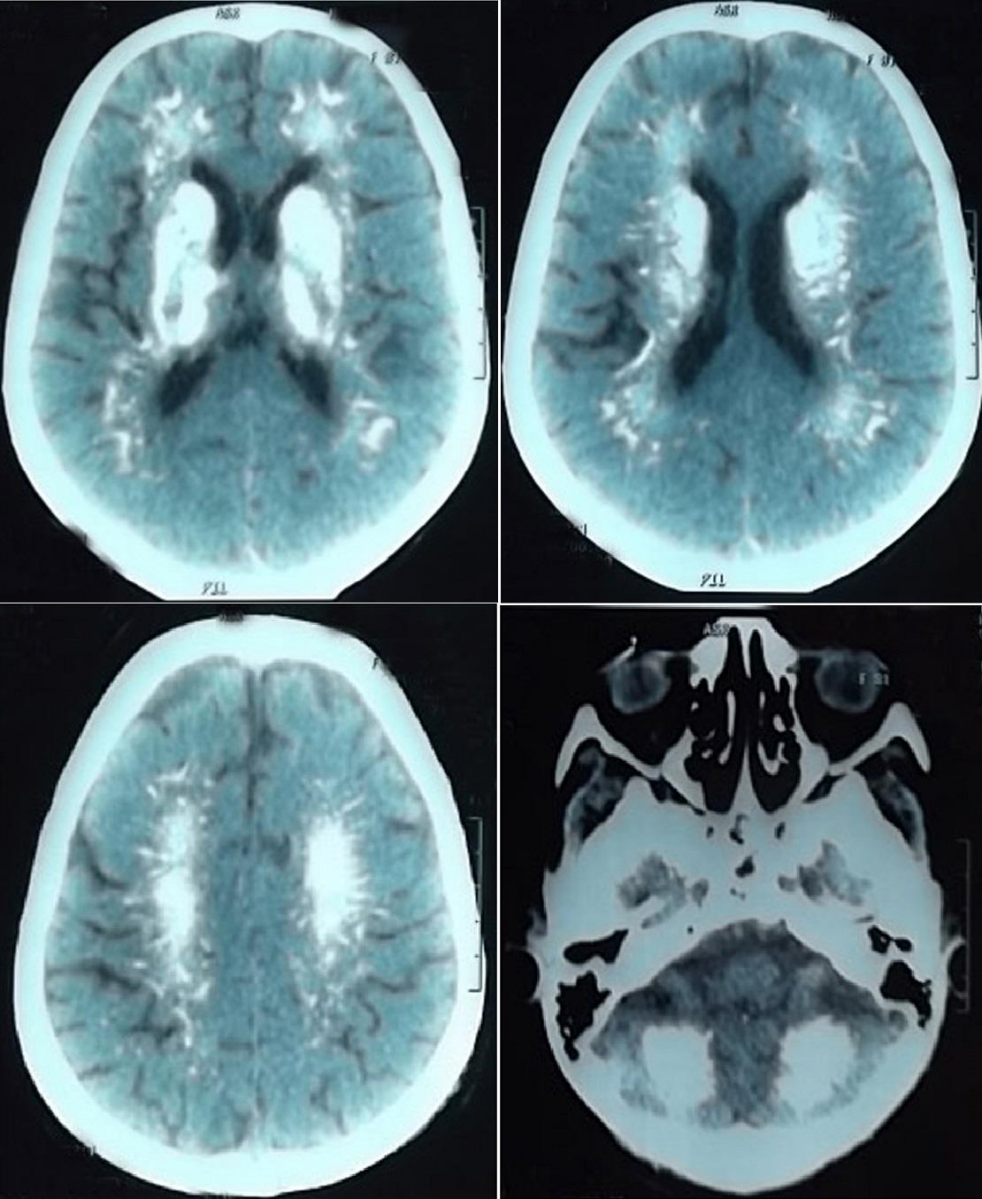
Calcifications étendues des noyaux gris centraux, à la jonction substance blanche substance grise linéaires, du bulbe cérébral et du cervelet

